# Crystal-Size Effects on Carbon Dioxide Capture of a Covalently Alkylamine-Tethered Metal-Organic Framework Constructed by a One-Step Self-Assembly

**DOI:** 10.1038/srep19337

**Published:** 2016-01-13

**Authors:** Yun Kyeong Kim, Sung-min Hyun, Jae Hwa Lee, Tae Kyung Kim, Dohyun Moon, Hoi Ri Moon

**Affiliations:** 1 Department of Chemistry, Ulsan National Institute of Science and Technology (UNIST), 50 UNIST-gil, Ulsan 44919, Republic of Korea; 2 Beamline Division, Pohang Accelerator Laboratory, 80 Jigokro-127-beongil, Nam-gu, Pohang, Gyungbuk 37673, Republic of Korea

## Abstract

To enhance the carbon dioxide (CO_2_) uptake of metal-organic frameworks (MOFs), amine functionalization of their pore surfaces has been studied extensively. In general, amine-functionalized MOFs have been synthesized via post-synthetic modifications. Herein, we introduce a one-step construction of a MOF ([(NiL_ethylamine_)(BPDC)] = **MOF**_**NH2**_; [NiL_ethylamine_]^2+^ = [Ni(C_12_H_32_N_8_)]^2+^; BPDC^2−^ = 4,4‘-biphenyldicarboxylate) possessing covalently tethered alkylamine groups without post-synthetic modification. Two-amine groups per metal centre were introduced by this method. **MOF**_**NH2**_ showed enhanced CO_2_ uptake at elevated temperatures, attributed to active chemical interactions between the amine groups and the CO_2_ molecules. Due to the narrow channels of **MOF**_**NH2**_, the accessibility to the channel of CO_2_ is the limiting factor in its sorption behaviour. In this context, only crystal size reduction of **MOF**_**NH2**_ led to much faster and greater CO_2_ uptake at low pressures.

Global warming by the accumulation of carbon dioxide (CO_2_) is a serious environmental problem, because it leads to severe natural disasters^1^. Therefore, the need for practical CO_2_ adsorbents is critical, and various efforts have been made in this field^2^,^3^,^4^. Currently, alkylamine solutions are used as CO_2_ adsorbents in industry due to their ease of use and low cost^5^. However, ease of degradation, toxic by-products, and a high regeneration energy are endemic problems of this technology^6^. Thus, tethering alkylamine groups on solid supports is considered as a useful strategy to stabilize the amine groups and reduce the energy required for regeneration^4^,^7^,^8^,^9^,^10^,^11^,^12^. Metal-organic frameworks (MOFs) provide appropriate solid supports due to the chemical tunability of their metal building blocks and organic ligands. To prepare amine-functionalized MOFs, open-metal sites (OMSs) on the internal pore surfaces are decorated with multi-amine molecules to capture CO_2_ molecules^13^,^14^,^15^. Physical impregnation of polyalkylamines like polyethyleneimine (PEI) into pore-activated MOFs is also the good approach to introduce active amine groups into the pore^16^. These synthetic methods allow the MOFs to show advanced CO_2_ uptake behaviour. However, several preparation steps and leaching of the impregnated amines during multiple regeneration cycles can be potential problems in their practical utilization. Thus, covalent grafting of amine groups on the organic building blocks is very efficient in achieving stable amine-functionalized solid adsorbents^17^,^18^,^19^. However, amino groups substituted to aromatic ligands do not interact strongly with CO_2_ due to the electron withdrawing effect of benzene rings^16^,^20^. Thus, herein we suggest a one-step self-assembly toward a MOF with a pore surface decorated with covalently tethered alkylamines, which can be stable during regeneration. To achieve this synthetic approach, we selected a Ni(II)-complexed macrocycle as a metal building block, which contains two pendant ethylamine groups ([NiL_ethylamine_]^2+^ = [Ni(C_12_H_32_N_8_)]^2+^)^21^ (Fig. 1a). The amine-tethered MOF shows chemical interactions with CO_2_ leading to the formation of ammonium carbamate, which is elucidated by infrared (IR) spectroscopy. In addition, only reducing the crystal size leads at most sevenfold enhancement of CO_2_ adsorption by increasing the gas accessibility, which resolves the diffusion resistance caused by the high amine density in the pore. Adsorption-desorption cyclic performance is also tested to show the durability of the MOF.

## Results and Discussion

### X-ray Structure of MOF_NH2_-as

**Figure 1:**
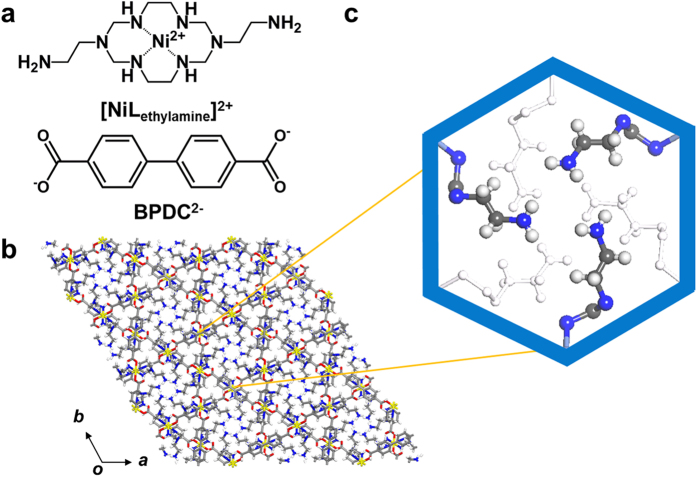
(**a**) Synthetic strategy, and (**b,c**) the structure (ab plane) of a covalently alkylamine-tethered MOF (**MOF_NH2_-as**). (Colour scheme: Ni, yellow; C, grey; O, red; N, blue).

Self-assembly of [NiL_ethylamine_]^2+^ and an organic ligand, BPDC^2−^ yields rod-shaped purple crystals of {[(NiL_ethylamine_)(BPDC)]·3H_2_O} (**MOF**_**NH2**_**-as**). Each Ni^II^ macrocycle has a square planar geometry and acts as a linear linker; its axial sites are coordinated by carboxylate anions from two different BPDC^2−^ ligands in a monodentate fashion, resulting in octahedral centres (Fig. S1). Infinite coordination between BPDC^2−^ and Ni^II^ macrocycles results in one dimensional (1D) chains, which are extended in three different directions. This was similar to a previously reported structure^22^, generating honeycomb-like 1D channels. The pendant ethylamine groups are exposed to the 1D channels, decorating the internal pore surface (Fig. 1b,c). Amine moieties from three pendant arms are adjacent to each other in the pore (the shortest distance between amine groups is 2.70 Å), generating a very narrow pore diameter of 1.59 Å. The void volume calculated by PLATON^23^ is 612.8 Å^3^ (9.1%).

### Stability of MOF_NH2_-as

**Figure 2:**
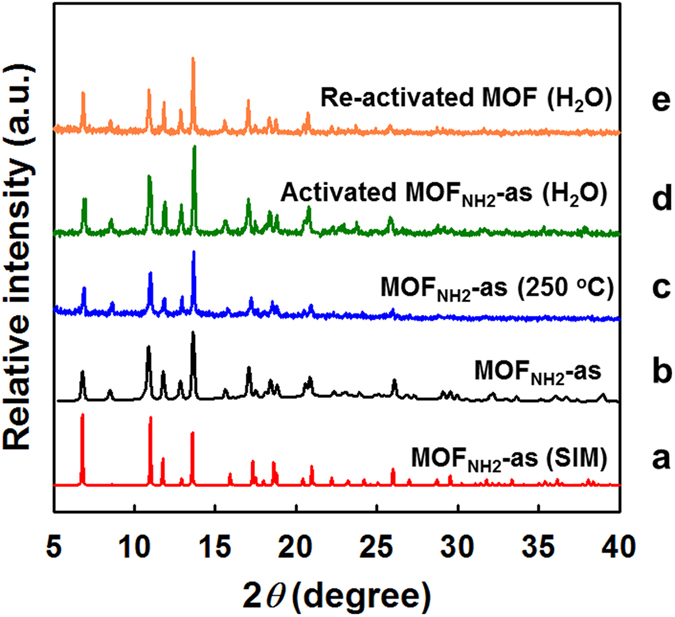
XRPD patterns of (**a**) simulation from single-crystal XRD data of MOF_NH2_-as, (b) MOF_NH2_-as, (c) MOF_NH2_-as after heating at 250 °C and (**d**) the activated MOF after immersing in water for 24 h (hydrated MOF). (**e**) Re-activated MOF of the hydrated MOF.

Thermogravimetric analysis (TGA) of **MOF**_**NH2**_**-as** (Fig. S2) revealed weight loss up to a temperature of ~230 °C, which was concurrent to the total weight percent of guest water molecules (8.6 wt%) occupying the void spaces. The X-ray powder diffraction (XRPD) pattern of as-synthesized **MOF**_**NH2**_**-as** showed strong reflections in the region 5−30 °, which were commensurated with the simulated pattern from single crystal X-ray diffraction data (Fig. 2a,b). After heating up to 250 °C under pure N_2_ flow, the MOF retained the same structure, showing high thermal stability (Fig. 2c). Since real flue gas usually contains more than 10% (v/v) water and water molecules are very good ligands for metal centres, the water stabilities of MOFs as well as the amine moieties coordinated to the OMSs cannot be assured under humid condition. In the present study, upon suspending the activated MOF in water for 24 h, its structure and the crystallinity were maintained as evidenced by the XRPD pattern (Fig. 2d). Re-activation of the hydrated MOF also resulted in the intact structure as shown in Fig. 2e. Therefore, **MOF**_**NH2**_**-as**, in which the amine moieties were tethered by covalent bonds, is a potentially good sorbent due to its thermal and water stability.

### Gas Sorption Properties of MOF_NH2_

**Figure 3:**
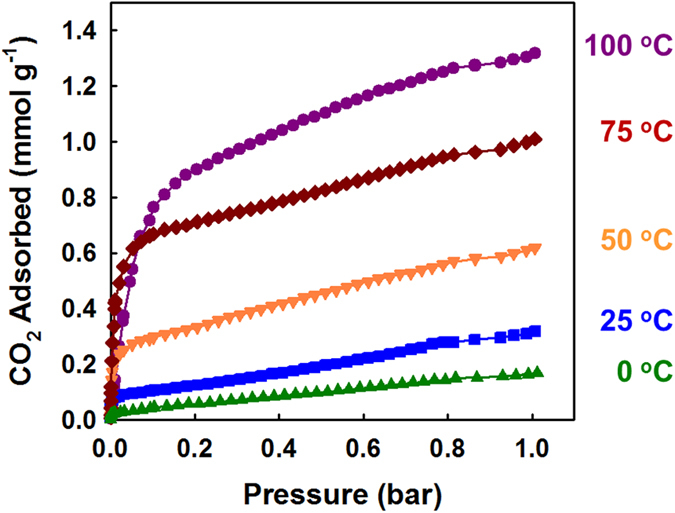
CO_2_ adsorption isotherms of **MOF**_**NH2**_ obtained at the various temperatures.

After activation of the **MOF**_**NH2**_**-as** crystals at 90 °C under vacuum for 7 h (**MOF**_**NH2**_), **MOF**_**NH2**_ maintained the same structure to **MOF**_**NH2**_**-as** as evidenced by the XRPD patterns (Fig. S3). N_2_ isotherm of **MOF**_**NH2**_ at 77 K showed a type II shape, indicating that the narrow channels (1.59 Å) are not accessible to N_2_ molecules (3.64 Å) (Fig. S4). The CO_2_ adsorption isotherms for **MOF**_**NH2**_ at several temperatures are depicted in Fig. 3. At 0 °C and 1 bar, **MOF**_**NH2**_ adsorbed a small amount of CO_2_ (0.17 mmol g^−1^). However, as the temperature increased to 100 °C, the CO_2_ uptake increased to 1.32 mmol g^−1^ (Table S3). This behaviour is the opposite of what has been observed for previous amine-grafted MOFs, which show a decrease in CO_2_ uptake with increasing temperature^14^,^15^. In the previous reports, owing to their large pores, chemisorption was followed by physisorption, which might be weakened at the higher temperature. On the other hand, **MOF**_**NH2**_ does not have a large enough pore diameter to access multi-layer adsorption, and thus the uptake by physisorption can be exclusively considered. Therefore, at high temperatures the chemisorption in **MOF**_**NH2**_ is much sufficiently occurred to form C-N bonds between amines and CO_2_. The desorption isotherms at 0–75 °C did not trace back to the adsorption curve with large hysteresis even in the low-pressure region, and at 100 °C the adsorbed CO_2_ molecules were completely desorbed by evacuation (Figs S5 and S6). These different desorption behaviours upon temperatures were verified by the gravimetric uptake result under 100% CO_2_ gas flow (Fig. S7). It was found that adsorption and desorption are at the equilibrium around 100  °C, and thus the chemisorbed CO_2_ molecules in **MOF**_**NH2**_, can be liberated at 100 °C upon pressure reduction.

As described previously, the pore size of **MOF**_**NH2**_ is only half of the kinetic diameter of CO_2_ (3.3 Å). Then, how the CO_2_ adsorption can occur in this MOF? The flexible movement of the pendant alkylamine groups may allow the uptake of CO_2_. At the low temperature, 100 K, at which X-ray single crystal data was collected, the alkylamine groups did not show significant thermal disorderness. However, as the temperature increases, thermal motion of the alkylamines, which have conformational flexibility, become active. Consequently, at certain moments the thermal disorderness makes the pores accessible to CO_2_ molecules. Therefore, higher temperatures afford not only stochastically more possibilities to generate a suitable pore size, but also thermal energy to produce chemical bonds between the amino groups and CO_2_.

### Crystal Size Effects on CO_2_ Uptake of MOF_NH2_

**Figure 4:**
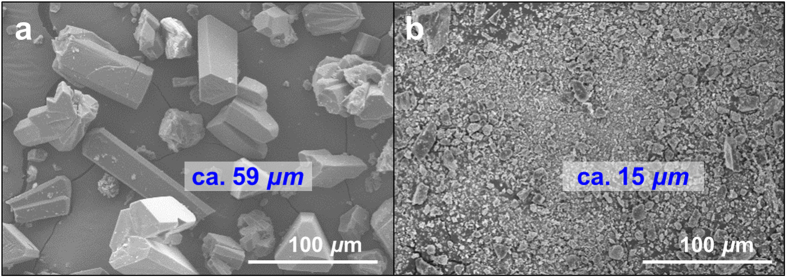
Scanning electron microscope (SEM) images of (**a**) as-synthesized MOF_NH2_ crystals (MOF_NH2:crystal_), and (**b**) MOF_NH2_ after grinding (MOF_NH2:powder_).

**Figure 5:**
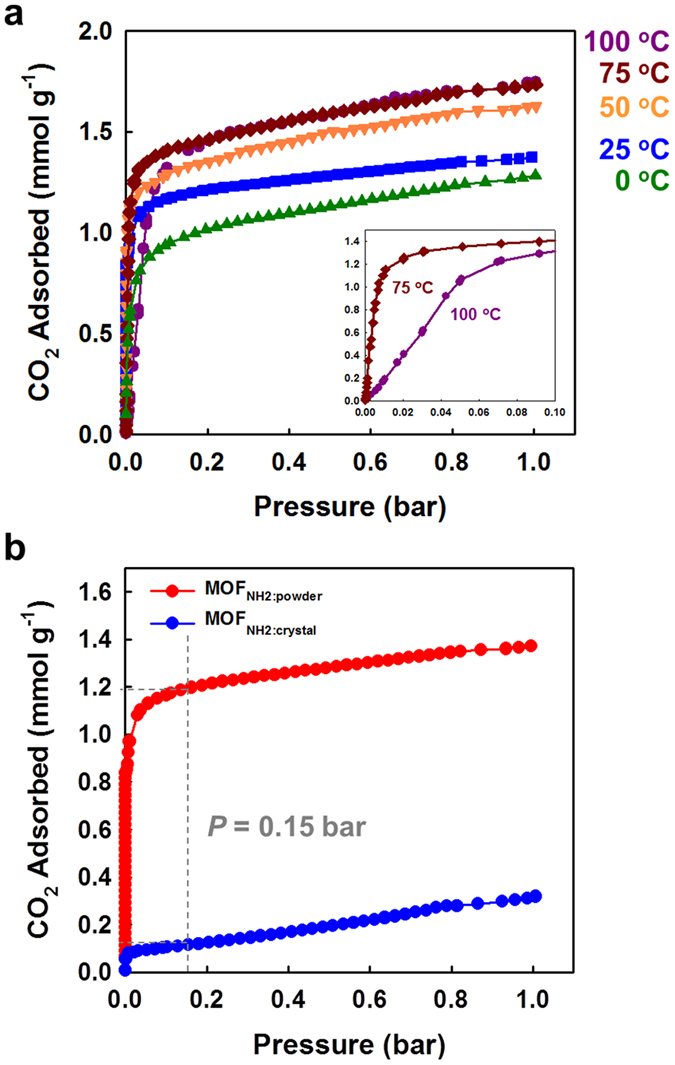
CO_2_ adsorption isotherms of (**a**) MOF_NH2:powder_ obtained at the various temperatures. (**b**) Comparison of the CO_2_ adsorption behaviour of MOF_NH2:crystal_ and MOF_NH2:powder_ at 25 °C.

Based on this understanding, the access of CO_2_ into the MOF is of critical importance. Accordingly, we compared the CO_2_ sorption behaviours of **MOF**_**NH2**_ with the different crystal size, **MOF**_**NH2:crystal**_, which have been treated so far, and well-ground **MOF**_**NH2**_ powder (**MOF**_**NH2:powder**_). **MOF**_**NH2:powder**_ was prepared by pulverizing **MOF**_**NH2:crystal**_, using a sample grinder with stainless steel vial and ball. As shown in the scanning electron microscope (SEM) images (Fig. 4), **MOF**_**NH2:crystal**_ are several tens of micrometres long (average 58.7 ± 27.8 μm measured from 71 crystals), whilst the size of **MOF**_**NH2:powder**_(average 15.4 ± 12.0 μm measured from 90 crystals) is mainly distributed in a few micrometre range. Interestingly, the only reduction in crystal size of **MOF**_**NH2**_ resulted in much faster and greater CO_2_ adsorption (Fig. 5a, S8, and Table S3). The uptake of **MOF**_**NH2:powder**_ at 1 bar were greater by a factor of 1.3–7.5 than those of **MOF**_**NH2:crystal**_ over all temperatures. This might be attributed to a decrease of the channel length in each grain of the adsorbent, which results in high accessibility under the same condition. In contrast with **MOF**_**NH2:crystal**_, **MOF**_**NH2:powder**_ adsorbed the almost same CO_2_ uptake at 75 and 100 °C (~7.6 wt%). This suggests that under the guaranteed condition of the CO_2_ accessibility, the uptake of ~1.75 mmol g^−1^ might be the maximum value in this MOF. The relatively slow kinetics was observed at 100 °C because the adsorption and desorption are at the equilibrium around 100 °C as mentioned previously (Fig. 5a inset, and S9). At 0.15 bar and 25 °C, comparable to the CO_2_ partial pressure of a typical post-combustion flue gas^24^, the CO_2_ uptake of **MOF**_**NH2:powder**_and **MOF**_**NH2:crystal**_ are 1.19 and 0.12 mmol g^−1^, respectively (Fig. 5b). In other words, the sorption ability at 0.15 bar improved tenfold with a decrease in crystal size. Our result shows the good agreement with that of the amine-modified mesoporous silicas. Sayari group reported the effect of the pore length on CO_2_ adsorption in the porous silica with high loading of PEI^25^. SBA-15PLT silica with very short pore channels showed much enhanced adsorption and desorption kinetics as well as low temperature CO_2_ uptake. It is obvious that high amine loading is necessarily required for high CO_2_ uptake. However, it always comes with diffusion limitation, and thus, the optimization of the loading amount ought to be accompanied. In this context, the reduction of the pore length can be the simple but effective way to enhance the sorption behaviour, and our study is the first report of this trial using MOFs.

### Evidence of Chemical Interaction between MOF_NH2:powder_ and CO_2_

**Figure 6:**
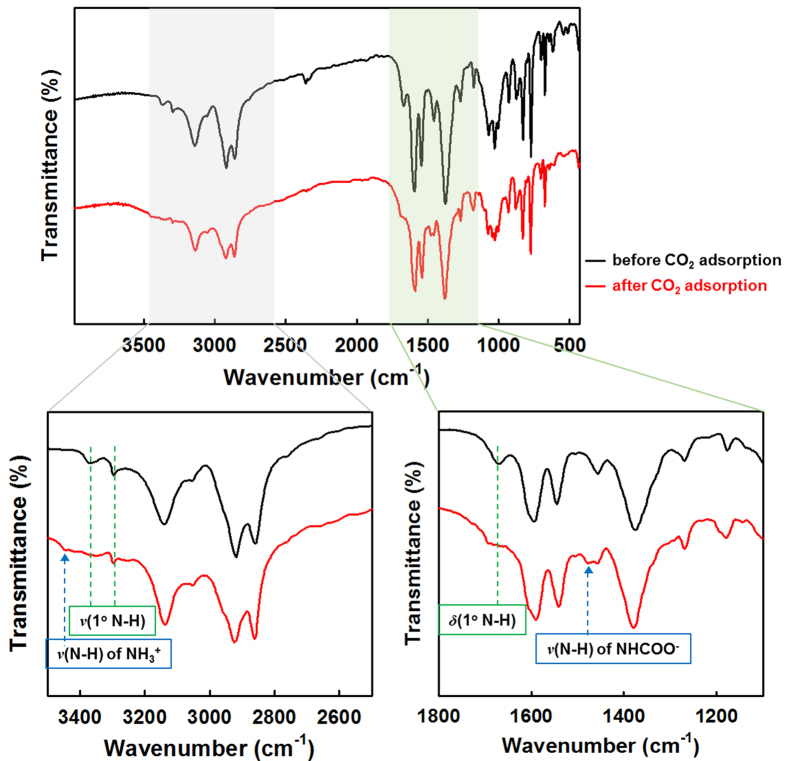
Infrared spectra for MOF_NH2:powder_ before (black) and after (red) adsorbing CO_2_.

To verify the chemical interaction between CO_2_ molecules and the pendant amine groups, the IR spectra of **MOF**_**NH2:powder**_ before and after CO_2_ adsorption were compared (Fig. 6). Upon CO_2_ adsorption at 75 °C, the stretching bands at 3368, 3293 cm^−1^ and bending band at 1660 cm^−1^, which correspond to the primary amine (N-H) tethered to the MOF mostly disappeared, remaining the small trace. Simultaneously, new peaks were observed at 3444 cm^−1^ and 1478 cm^−1^, assigned to N-H stretching of carbamate (NHCOO^−^) group and NH_3_^+^ deformation, respectively^26^,^27^. This result indicated that the chemical interactions between CO_2_ and **MOF**_**NH2:powder**_ form ammonium carbamate, which results from a 2:1 amine:CO_2_ stoichiometric reaction (equation (1)).


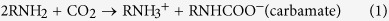


The secondary amine (N-H) peak from the Ni^II^ macrocycle at 3135 cm^−1^ was maintained, showing that CO_2_ reacts with only the primary amine of the pendant in the MOF. A 600 MHz ^13^C NMR analysis also supports the formation of carbamate as a result of the chemical interaction between amine groups and CO_2_ molecules (Fig. S10). Unlike the spectra of **MOF**_**NH2**_ before adsorption and after regeneration, that of **MOF**_**NH2**_ adsorbing CO_2_ molecules show the distinct peak at 160.5 ppm. Because a carbamic acid C = O resonance is usually observed at 157–160 ppm and carbamate at 164–165 ppm^28^,^29^, the obseved peak might be the result of the equilibrium between carbamate and carbamic acid groups through proton transfer in the solution state. The formed carbamate groups can block the narrow pore to prevent the diffusion of the next CO_2_ molecules. However, from the maximum CO_2_ uptake amount, the pendants terminated by carbamate or ammonium groups might be expected to have the flexible motions to provide the accessible pathway to CO_2_ molecules including the conformational changes of alkyl chains. The isosteric heat of adsorption (*Q*_st_) by using CO_2_ adsorption data at 50 and 75 °C was calculated by applying a single site Langmuir-Freundlich model (Figs S11 and S12), because the chemisorption is major contribution to the total uptake at those temperatures. *Q*_st_ is −59.5 kJ mol^−1^ at zero coverage, which is reasonable but relatively small value for the chemisorbed CO_2_ in MOF.

### CO_2_ Cyclic Performance for MOF_NH2:powder_

**Figure 7:**
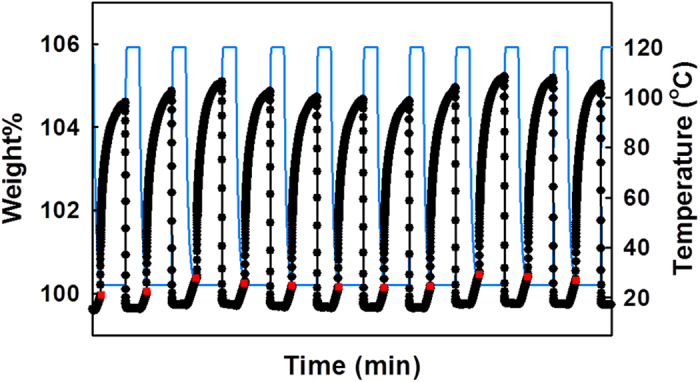
Adsorption-desorption cyclic performance for MOF_NH2:powder_, showing reversible uptake from simulated flue gas (15% CO_2_ balanced with N_2_). CO_2_ was introduced at the red points.

Since durability of CO_2_ adsorbents is very important for practical applications, adsorption-desorption cyclic performance of **MOF**_**NH2:powder**_ was tested by using TGA with a combined temperature swing and nitrogen purge approach (Fig. 7). The sample was activated at 150 °C under pure N_2_ flow for 7 h. Then, a simulated flue gas was introduced into the furnace for 2 h at 25 °C, followed by regeneration at 120 °C for 1 h under pure N_2_ gas. A CO_2_ uptake capacity of 4.80 wt% (1.09 mmol g^−1^) was recorded on an average over 12 cycles, and there was no decrease in CO_2_ uptake, indicating that the CO_2_ adsorption ability of **MOF**_**NH2:powder**_ is maintained over repeated cycling.

## Conclusions

In conclusion, an amine-functionalized MOF, **MOF**_**NH2**_, was successfully synthesized via a one-step construction method. The structure of **MOF**_**NH2**_ was determined by using single-crystal XRD, confirming that the covalently tethered alkylamine groups conserved the loading of amine groups as two per metal centre. **MOF**_**NH2**_ showed enhanced CO_2_ adsorption as temperature increases, and the results of sorption experiments and IR spectroscopy revealed that chemisorption occurred through the interaction between CO_2_ molecules and amine groups. Since the narrow channel in the MOF restricts the easy access of CO_2_ molecules, reducing the crystal size led faster and greater CO_2_ adsorption under same sorption condition with diminished diffusion resistance and enhanced amine accessibility inside the pores. The chemical interaction between the primary amine groups tethered to the MOF and CO_2_ molecules formed ammonium carbamate, which was reversibly dissociated at mild temperature, 100 °C, to release CO_2_ molecules. **MOF**_**NH2**_ was found to be a renewable CO_2_ adsorbent with good stability over repeated cycling.

## Methods

### Materials and Methods

All chemicals and solvents used in the syntheses were of reagent grade and were used without further purification. [NiL_ethylamine_](ClO_4_)_2_ was prepared by a reported method with minor modifications^21^. Infrared spectra were measured on a Thermo Fisher Scientific Nicolet 6700 FT-IR spectrometer. Thermogravimetric analyses (TGA) were performed under N_2_ at a scan rate of 5 °C/min and under pure CO_2_ at a scan rate of 1 °C/min using a Q50 from TA instruments. XRPD data were collected using both a Bruker D2 PHASER automated diffractometer at 30 kV and 10 mA for Cu Kα (λ = 1.54050 Å), with a step size of 0.02 ° in 2θ and an ADSC Quantum-210 detector at 2D SMC with a silicon (111) double crystal monochromator (DCM) at the Pohang Accelerator Laboratory, Korea. Scanning electron microscope (SEM) images were taken using a Quanta 200 microscope (FEI) operating at 18 kV. The gas sorption data were collected by using a BELsorp-MAX. UV/Vis diffuse reflectance spectra were recorded on a Cary 5000 UV/Vis spectrophotometer. Nuclear magnetic resonance (NMR) spectra were recorded on a Varian VNMRS 600 spectrometer. Elemental analyses were conducted by UNIST Central Research Facilities centre (UCRF) in Ulsan National Institute of Science and Technology (UNIST).

### Synthesis of MOF_NH2_-as, {[(NiL_ethylamine_)(BPDC)]·3H_2_O}

[NiL_ethylamine_](ClO_4_)_2_ (0.04 g, 0.07 mmol) and Na_2_BPDC (0.02 g, 0.07 mmol) were dissolved in *N,N-*diethylformamide (4 mL) and in a mixed solution of acetonitrile (MeCN) and H_2_O (MeCN:H_2_O = 2 mL:1 mL), respectively. The solution of Na_2_BPDC was diffused onto the former solution and powder was formed at the boundary of the layered solution prior to the formation of crystals. The mixed solution allowed to stand at room temperature for 1 day until the pale purple crystals were formed along with some powder. Only crystals were used for analyses. Yield: 22.7%. FT-IR (KBr): 3368 and 3293 cm^−1^ (NH), 3062 cm^−1^ (CH), 1587 cm^−1^ and 1379 cm^−1^ (COO^−^); UV-Vis (diffuse reflectance spectrum): λ_max_ 517 nm (Ni^II^ d-d transition); Elemental analysis calcd., found for Ni_1_C_26_H_46_N_8_O_7_: C (48.69, 49.36), H (7.23, 7.10), N (17.47, 17.52).

### Preparation of MOF_NH2:crystal_ and MOF_NH2:powder_

The as-synthesized compounds, **MOF**_**NH2**_**-as**, were heated at 90 °C under vacuum for 7 h, and then cooled to an ambient temperature and refilled with Ar (**MOF**_**NH2:crystal**_). To prepare the powder samples (**MOF**_**NH2:powder**_), **MOF**_**NH2**_**-as** was pulverized for 10 s using a sample grinder with a stainless steel vial and ball (ShakIR sample grinder, PIKE), which is usually used for preparation of infrared spectroscopy samples. The resultant powder was also activated at 90 °C for 7 h, yielding **MOF**_**NH2:powder**_.

### Single-Crystal X-ray crystallography

Single-crystals of **MOF**_**NH2**_**-as** were coated with paratone-*N* oil because they lost their crystallinity upon exposure to the air. The diffraction data of **MOF**_**NH2**_**-as** were measured at 100 K using synchrotron employing a PLSII-2D SMC an ADSC Quantum-210 detector with a silicon (111) double crstal monochromator (DCM) at Pohang Accelerator Laboratory, Korea. The ADSC Q210 ADX program^30^ was used for both data collection, and HKL3000sm (Ver. 703r)^31^ was used for cell refinement, reduction and absorption correction. The structures of **MOF**_**NH2**_**-as** were solved using direct methods with SHELX-XS (Ver. 2008) and refined by full-matricx least-squares calculation with SHELX-XL (Ver. 2008) program package^32^. An half of ligands, an half of Ni ions and one unligated water molecule were observed as an asymmetric unit. For the structure **MOF**_**NH2**_**-as**, the alkyl amine pendant group was restrained using ISOR during the least-squares refinement. All non-hydrogen atoms in whole structures were refined anisotropically and hydrogen atoms were assigned geometrically using a riding model. Refinement of the structure **MOF**_**NH2**_**-as** converged at a final *R*_*1*_ = 0.0709, *wR*_*2*_ = 0.2208 for 18729 reflections with *I* > 2*σ* (*I*); *R*_*1*_ = 0.0776, *wR*_*2*_ = 0.2294 for all reflections. The largest difference peak and hole were 0.959 and −0.543 e·Å^−3^, respectively. A summary of the crystals and some crystallographic data are given in Table S1 and S2. CCDC 1044896 contains the supplementary crystallographic data. The data can be obtained free of charge at www.ccdc.cam.ac.uk/conts/retrieving.html or from the Cambridge Crystallographic Data Centre, 12, Union Road, Cambridge CB2 EX, UK.

## Additional Information

**How to cite this article**: Kim, Y. K. *et al.* Crystal-Size Effects on Carbon Dioxide Capture of a Covalently Alkylamine-Tethered Metal-Organic Framework Constructed by a One-Step Self-Assembly. *Sci. Rep.* **6**, 19337; doi: 10.1038/srep19337 (2016).

## Supplementary Material

Supplementary Information
