# Supplementary material for: Crystal-Size Effects on Carbon Dioxide Capture of a Covalently Alkylamine-Tethered Metal-Organic Framework Constructed by a One-Step Self-Assembly
Source: Sci Rep. 2016 Jan 13;6:19337. doi: 10.1038/srep19337 (PMC4725930; doi:10.1038/srep19337)
Supplement: Supplementary Information [file srep19337-s1.pdf]

Supporting Information

Crystal-Size Effects on Carbon Dioxide Capture of a Covalently Alkylamine-Tethered Metal-Organic Framework Constructed by a One-Step Self-Assembly

Yun Kyeong Kim[1,+], Sung-min Hyun[1,+], Jae Hwa Lee[1], Tae Kyung Kim[1], Dohyun Moon[2], and Hoi Ri Moon[1,*]

[1]Department of Chemistry, Ulsan National Institute of Science and Technology (UNIST), 50 UNIST-gil, Ulsan 44919, Republic of Korea
[2]Beamline Division, Pohang Accelerator Laboratory 80 Jigokro-127-beongil, Nam-gu, Pohang, Gyungbuk 37673, Republic of Korea
[*] Corresponding Author, e-mail: hoirimoon@unist.ac.kr
[+] these authors contributed equally to this work

Experimental Section

Materials and methods

All chemicals and solvents used in the syntheses were of reagent grade and were used without further purification. $[NiL_{ethylamine}](ClO_4)_2$ was prepared by a reported method with minor modifications.[S1] Infrared spectra were measured on a Thermo Fisher Scientific Nicolet 6700 FT-IR spectrometer. Thermogravimetric analyses (TGA) were performed under N_2 at a scan rate of 5 ^oC/min and under pure CO_2 at a scan rate of 1 ^oC/min using a Q50 from TA instruments. XRPD data were collected using both a Bruker D2 PHASER automated diffractometer at 30 kV and 10 mA for Cu Kα (λ = 1.54050 Å), with a step size of 0.02^o in 2θ and an ADSC Quantum-210 detector at 2D SMC with a silicon (111) double crystal monochromator (DCM) at the Pohang Accelerator Laboratory, Korea. Scanning electron microscope (SEM) images were taken using a Quanta 200 microscope (FEI) operating at 18 kV. The gas sorption data were collected by using a BELsorp-MAX. UV/Vis diffuse reflectance spectra were recorded on a Cary 5000 UV/Vis spectrophotometer. Nuclear magnetic resonance (NMR) spectra were recorded on a Varian VNMRS 600 spectrometer. Elemental analyses were conducted by UNIST Central Research Facilities centre (UCRF) in Ulsan National Institute of Science and Technology (UNIST).

Synthesis of $[NiL_{ethylamine}](ClO_4)_2$ ($[Ni(C_{12}H_{32}N_8)](ClO_4)_2$).[S1] $Ni(OAc)_2{\cdot}4H_2O$ (9.0 g, 0.04 mol) was dissolved in methanol (50 mL) and placed in an ice-bath, and 99% ethylenediamine (13.5 mL, 0.20 mol) was added dropwisely to it. Paraformaldehyde (6.1 g, 0.20 mol) was then added and dissolved with stirring at room temperature. Then, the reaction mixture was refluxed for 12 h. After 12 h reaction, the mixture solution was placed in an ice-bath and $HClO_4$ was added very slowly to pH 5~6 with stirring, and excess amount of $NaClO_4$ (ca. 2 g) was dissolved in the solution. The mixture solution was kept in room temperature over 1 week until the yellow powder was precipitated. The precipitates were filtered and reduced by triethylamine (TEA) solution in acetonitrile (MeCN) solvent. The final product was filtered and dried under vacuum. Yield: ~ 23%. FT-IR (KBr): 3304 and 3225 cm^{-1} (NH), 1093 cm^{-1} (ClO_4^-); UV/Vis (diffuse reflectance spectrum): λ_{max} 450 nm; Elemental analysis calcd., found for $Ni_1C_{12}H_{32}N_8O_8Cl_2$: C (26.40, 26.73), H (5.91, 5.89), N (20.52, 20.46).

**Synthesis of Sodium 4,4`-biphenyldicarboxylate ($Na_2BPDC$).** 4,4`-biphenyldicarboxylic acid (H_2BPDC) (2.42 g, 0.01 mol) was dispersed in water (25 mL) by stirring with a magnetic stirrer. NaOH solution, which is prepared through dissolving NaOH (1.20 g, 0.03 mol) in 7 mL water, was added to H_2BPDC mixture solution then the white powder was dissolved and small amount of solids were appeared soon. After the solution was filtered, ethanol (20 mL) was put into the solution and the solution was kept in refrigerator over 3 hours in order to help further precipitation of products. The white powder was filtered, washed with Ethanol, and dried by evacuation under air. Yield: ~72.8%.

Synthesis of MOF_{NH2}-as, $\{[(NiL_{ethylamine})(BPDC)]{\cdot}3H_2O\}$. $[NiL_{ethylamine}](ClO_4)_2$ (0.04 g, 0.07 mmol) and Na_2BPDC (0.02 g, 0.07 mmol) were dissolved in *N,N*-diethylformamide (4 mL) and in a mixed solution of acetonitrile (MeCN) and H_2O (MeCN:H_2O = 2 mL:1 mL), respectively. The solution of Na_2BPDC was diffused onto the former solution and powder was formed at the boundary of the layered solution prior to the formation of crystals. The mixed solution allowed to stand at room temperature for 1 day until the pale purple crystals were formed along with some powder. Only crystals were used for analyses. Yield: 22.7 %. FT-IR (KBr): 3363 and 3291 cm^{-1} (NH), 3062 cm^{-1} (CH), 1587 cm^{-1} and 1379 cm^{-1} (COO^-); UV-Vis (diffuse reflectance spectrum): λ_{max} 517 nm (Ni^{II} d-d transition); Elemental analysis calcd., found for $Ni_1C_{26}H_{46}N_8O_7$: C (48.69, 49.36), H (7.23, 7.10), N (17.47, 17.52).

Preparation of $MOF_{NH2:crystal}$ and $MOF_{NH2:powder}$. The as-synthesized compounds, **MOF_{NH2}-as,** were heated at 90 ^oC under vacuum for 7 h, and then cooled to an ambient temperature and refilled with Ar (**$MOF_{NH2:crystal}$**). To prepare the powder samples (**$MOF_{NH2:powder}$**), **MOF_{NH2}-as** was pulverized for 10 s using a sample grinder with a stainless steel vial and ball (ShakIR sample grinder, PIKE), which is usually used for preparation of infrared spectroscopy samples. The resultant powder was also activated at 90 ^oC for 7 h, yielding **$MOF_{NH2:powder}$**. FT-IR (KBr): 3368 and 3293 cm^{-1} (NH), 3055 cm^{-1}

(CH), 1594 and 1376 cm^{-1} (COO^-); UV-Vis (diffuse reflectance spectrum): λ_{max} 512 nm; Elemental analysis calcd., found for $Ni_1C_{26}H_{40}N_8O_4$: C (53.17, 52.47) H (6.86, 6.91) N (19.08, 18.78).

Synchrotron XRPD measurment. For **MOF_{NH2}-as** and **MOF_{NH2}**, the XRPD patterns were measured at 100 K with synchrotron radiation (λ = 1.20029 and 1.20024 Å, respectively). The mother liquor of only **MOF_{NH2}-as** crystals were decanted by filtration, and the resulting solids were finely ground and packed in capillary (diameter, 0.3 mm; wall thickness, 0.01 mm). To prepare the sample **MOF_{NH2}**, the filtered crystals of **MOF_{NH2}-as** were dried at 90 ^{o}C under vacuum for 7 h, they were finely ground and packed in capillary under Ar atmosphere in glove box (diameter, 0.3 mm; wall thickness, 0.01 mm). The diffraction data were measured with transmission-mode as Debye-Scherrer Pattern with the 180 mm of sample-to-detector distance in 60 s exposure on an ADSC Quantum-210 detector at 2D SMC with a silicon (111) double crystal monochromator (DCM) at the Pohang Accelerator Laboratory, Korea. The ADX program[S2] was used for data collection, and the Fit2D[S3] program was used for conversion of integrated 2D to 1D patterns, for wavelength and detector distance refinement and for a calibration measurement of a NIST Si 640c standard sample.

Single-Crystal X-ray crystallography. Single-crystals of **MOF_{NH2}-as** were coated with paratone-*N* oil because they lost their crystallinity upon exposure to the air. The diffraction data of **MOF_{NH2}-as** were measured at 100 K using synchrotron employing a PLSII-2D SMC an ADSC Quantum-210 detector with a silicon (111) double crstal monochromator (DCM) at Pohang Accelerator Laboratory, Korea. The ADSC Q210 ADX program[S2] was used for both data collection, and HKL3000sm (Ver. 703r)[S4] was used for cell refinement, reduction and absorption correction. The structures of **MOF_{NH2}-as** were solved using direct methods with SHELX-XS (Ver. 2008) and refined by full-matricx least-squares calculation with SHELX-XL (Ver. 2008) program package.[S5] An half of ligands, an half of Ni ions, and one unligated water molecule were observed as an asymmetric unit. For the structure **MOF_{NH2}-as**, the alkylamine pendant group was restrained using ISOR during the least-squares refinement. All non-hydrogen atoms in whole structures were refined anisotropically and hydrogen atoms were assigned geometrically using a riding model. Refinement of the structure **MOF_{NH2}-as** converged at a final R_1 = 0.0709, wR_2 = 0.2208 for 18729 reflections with $I > 2\sigma(I)$; R_1 = 0.0776, wR_2 = 0.2294 for all reflections. The largest difference peak and hole were 0.959 and -0.543 $e \cdot Å^{-3}$, respectively. A summary of the crystals and some crystallographic data are given in Table S1 – S2. CCDC 1044896 contain the supplementary crystallographic data. The data can be obtained free of charge at www.ccdc.cam.ac.uk/conts/retrieving.html or from the Cambridge Crystallographic Data Centre, 12, Union Road, Cambridge CB2 EX, UK.

Gas Sorption Study. The gas sorption isotherms were measured by using BELsorp-MAX. **$MOF_{NH2:crystal}$** was prepared after heating **MOF_{NH2}-as** at 90 ^{o}C under vacuum for 7 h and **$MOF_{NH2:powder}$** was prepared after heating pulverized **MOF_{NH2}-as** at 90 ^{o}C under for 7 h. After both

samples were introduced into the gas sorption apparatus, the resultant powder was additionally activated at 120 ^oC for 1 h and the gas sorption isotherms were measured. The N_2 for desolvated solids were monitored at 77 K. CO_2 gas sorption isotherms were measured at 0, 25, 50, 75, and 100 ^oC.

^{13}C NMR Analysis. To verify the chemical interaction between **$MOF_{NH2:powder}$** and CO_2 molecules, FT-NMR measurement was performed for three samples, **MOF_{NH2}-as**, **$MOF_{NH2:powder}$** after CO_2 adsorption at 75 ^oC, and its re-activated MOF. For measurement, the samples dispersed in dimethyl sulfoxide-d_6 were digested by adding the D_2O with DCl.

S1 Kang, S. G., Ryu, K., Jung, S. K. & Kim, J. Template synthesis, crystal structure, and solution behavior of a hexaaza macrocyclic nickel(II) complex containing two N-aminoethyl pendant arms. *Inorganica Chimica Acta.* **293**, 140-146 (1999).

S2 A. J. Arvai and C. Nielsen, ADSC Quantum-210 ADX Program; Area Detector System Corporation: Poway, CA, USA, (1983).

S3 Fit2D program:Andy Hammersley (E-mail: hammersley@esrf.fr), ESRF; 6 RUE JULES HOROWITZBP 22038043 GRENOBLE CEDEX 9FRANCE.

S4 Z. Otwinowski, W. Minor and C. W. Carter Jr, Sweet (Eds.), R. M. Methods in Enzymology 276 Part A; Academic Press: New York, 307 (1997).

S5 G. M. Sheldrick, SHELXTL-PLUS: Crystal Structure Analysis Package; Bruker Analytical X-Ray: Madison, WI, USA (1997).

Table S1. X-ray crystallographic data of **MOF_{NH2}-as**.

Compound	**MOF_{NH2}-as**
formula	$Ni_1C_{26}H_{40}N_8O_6$
crystal system	*Trigonal*
space group	*R*-3
fw	619.37
a, Å	26.077(4)
b, Å	26.077(4)
c, Å	11.485(2)
α, deg	90
β, deg	90
γ, deg	120
V, $Å^3$	6763.5(19)
Z	9
ρ_{calcd}, g cm^{-3}	1.369
temp , K	100(2)
λ, Å	0.70000
μ, mm^{-1}	0.668
goodness-of-fit (F^2)	1.066
F(000)	2952
reflections collected	18729
independent reflections	3250 [*R*(int) = 0.0283]
completeness to θ_{max}, %	94.7%
data/parameters/restraints	3250 / 188 / 12
θ range for data collection, deg	2.93 to 27.00
diffraction limits (*h*, *k*, *l*)	$-33 \leq h \leq 33$, $-33 \leq k \leq 33$, $-13 \leq l \leq 13$
refinement method	Full-matrix least-squares on F^2
R_1, wR_2 [I>2σ(I)]	R_1 = 0.0709,[a] wR_2 = 0.2208[b]
R_1, wR_2 (all data)	R_1 = 0.0776,[a] wR_2 = 0.2294[b]
largest peak, hole, $eÅ^{-3}$	0.959, -0.543

[a]$R = \Sigma||Fo| - |Fc||/\Sigma|Fo|$. [b]$wR(F^2) = [\Sigma w(Fo^2 - Fc^2)^2/\Sigma w(Fo^2)^2]^{½}$ where $w = 1/[\sigma^2(Fo^2) + (0.1653P)^2 + (7.425)P]$, $P = (Fo^2 + 2Fc^2)/3$.

Table S2. Selected bond distances [Å] and angles [°] of **MOF_{NH2}-as (100 K)**.

Ni(1)-N(1A)#1	2.057(3)	Ni(1)-N(1A)	2.057(3)
Ni(1)-N(2A)#1	2.061(4)	Ni(1)-N(2A)	2.061(4)
Ni(1)-O(1B)#1	2.1085(18)	Ni(1)-O(1B)	2.1085(18)
N(1A)#1-Ni(1)-N(1A)	180.0	N(2A)#1-Ni(1)-N(2A)	180.0
N(1A)#1-Ni(1)-N(2A)#1	94.03(18)	N(2A)#1-Ni(1)-N(1A)	85.97(18)
N(1A)#1-Ni(1)-N(2A)	85.97(18)	N(2A)-Ni(1)-N(1A)	94.03(18)
N(1A)#1-Ni(1)-O(1B)#1	93.79(10)	N(2A)#1-Ni(1)-O(1B)#1	88.56(11)
N(1A)#1-Ni(1)-O(1B)	86.21(10)	N(2A)#1-Ni(1)-O(1B)	91.44(11)
N(1A)-Ni(1)-O(1B)#1	86.21(10)	N(2A)-Ni(1)-O(1B)#1	91.44(11)
N(1A)-Ni(1)-O(1B)	93.79(10)	N(2A)-Ni(1)-O(1B)	88.56(11)
O(1B)#1-Ni(1)-O(1B)	180.0		

Symmetry transformations used to generate equivalent atoms:

#1 –x+1,-y,-z+1

Table S3. CO_2 uptake of **$MOF_{NH2:crystal}$** and **$MOF_{NH2:powder}$** at various temperatures (1 bar)

Adsorption temperature (°C)	$MOF_{NH2:crystal}$			$MOF_{NH2:powder}$		
	mmol g^{-1}	wt%	# of CO_2 per amine	mmol g^{-1}	wt%	# of CO_2 per amine
0	0.17	0.74	0.05	1.28	5.64	0.38
25	0.32	1.40	0.09	1.37	6.04	0.40
50	0.62	2.72	0.18	1.63	7.15	0.48
75	1.01	4.45	0.30	1.73	7.63	0.51
100	1.32	5.80	0.39	1.75	7.69	0.51

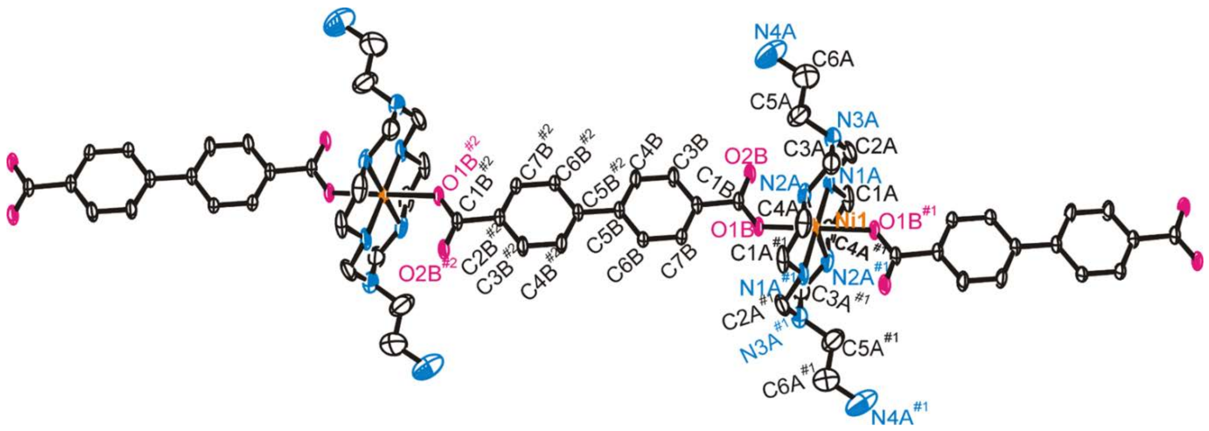

Figure S1. An ORTEP drawing of **MOF$_{NH2}$-as** at 100 K with an atomic numbering scheme (thermal ellipsoids at 30% probability).

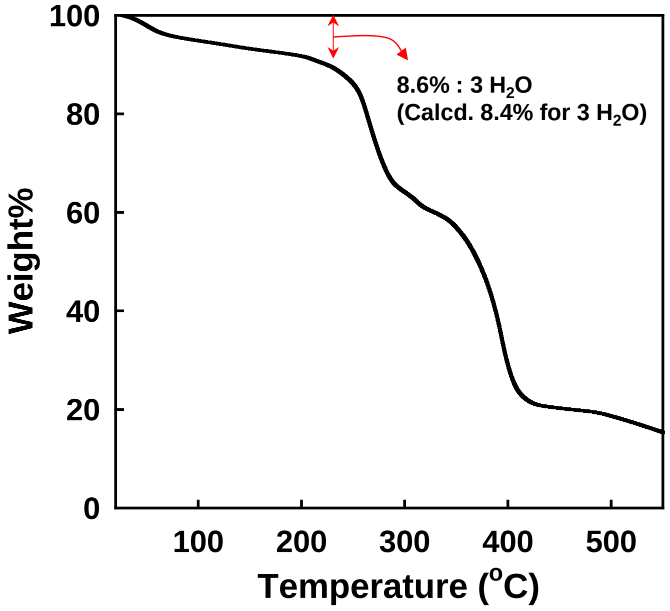

Figure S2. TGA trace of **MOF$_{NH2}$-as.**

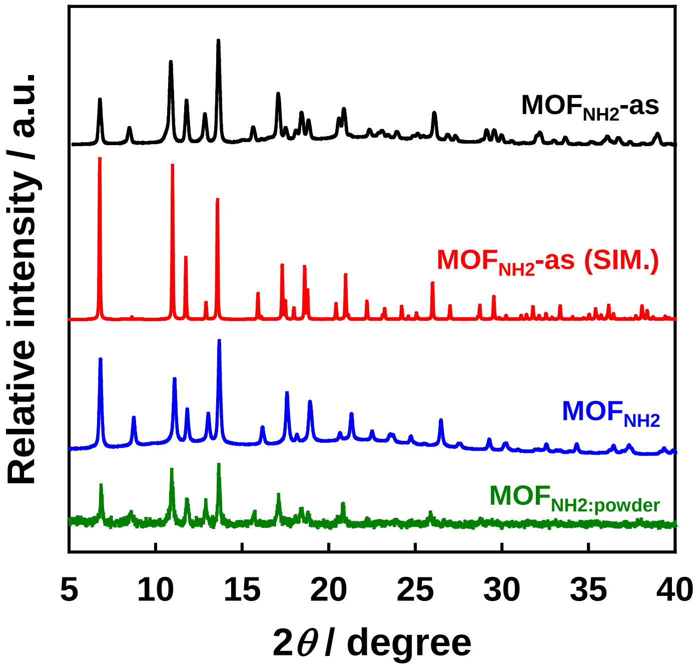

Figure S3. Comparison of simulated (red) and measured X-ray powder diffraction (XRPD) patterns of **MOF_{NH2}-as** (black), **MOF_{NH2}** (blue) and **$MOF_{NH2:powder}$** (green).

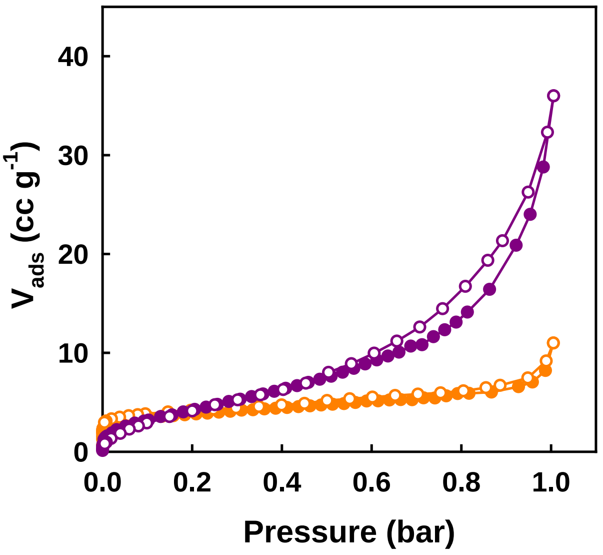

	$MOF_{NH2:crystal}$	$MOF_{NH2:powder}$
Surface area (m^2 g^{-1})	13.0	17.2
Pore volume (cm^3 g^{-1})	0.017	0.056

Figure S4. N_2 gas sorption isotherms of **$MOF_{NH2:crystal}$** (orange) and **$MOF_{NH2:powder}$** (purple) at 77 K**.**

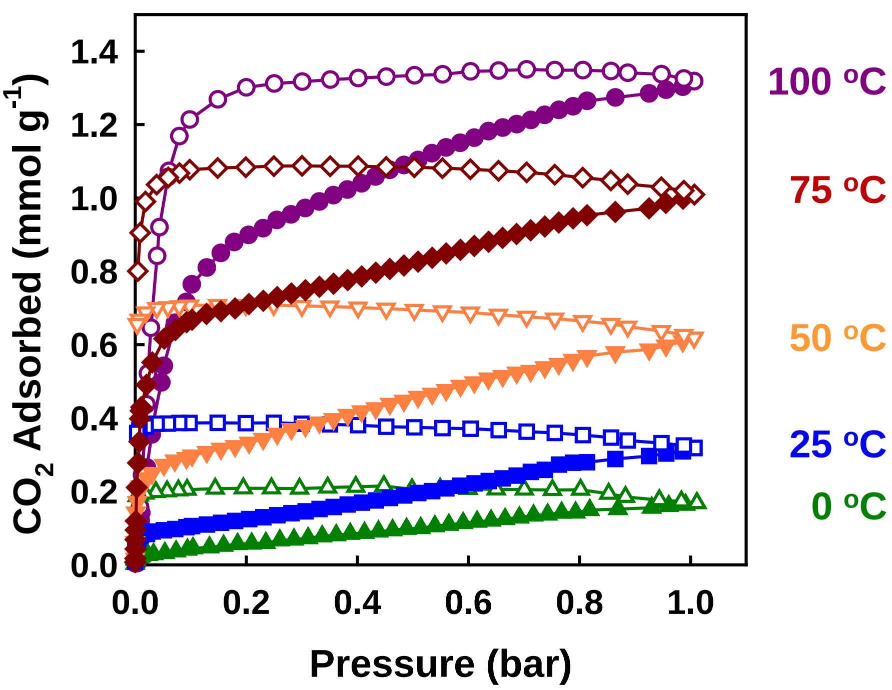

Figure S5. CO_2 gas sorption isotherms of **$MOF_{NH2:crystal}$** at 0-100 ^oC

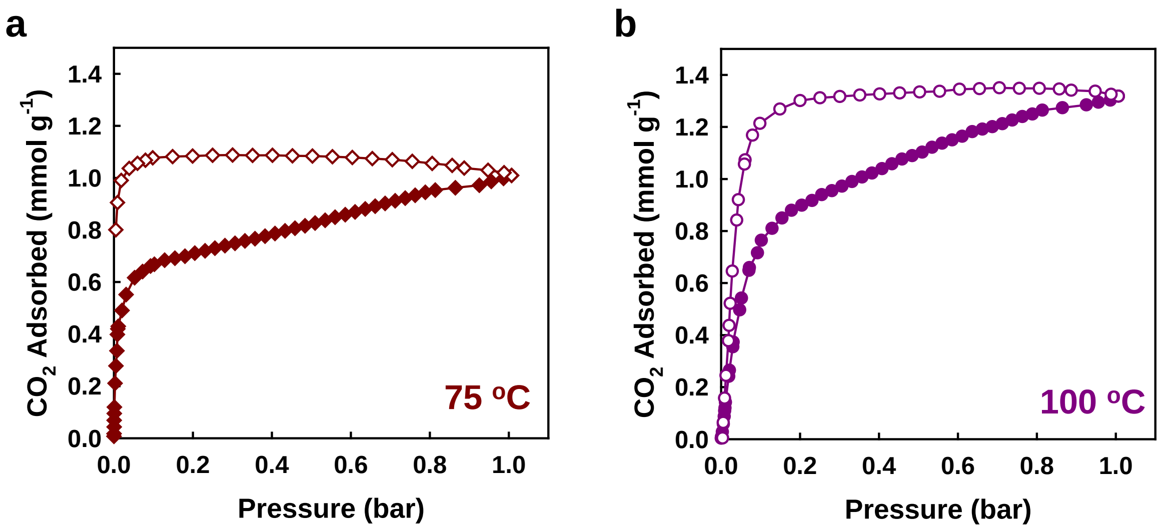

Figure S6. CO_2 gas sorption isotherms of **$MOF_{NH2:crystal}$** (a) 75 ^oC (b) at 100 ^oC

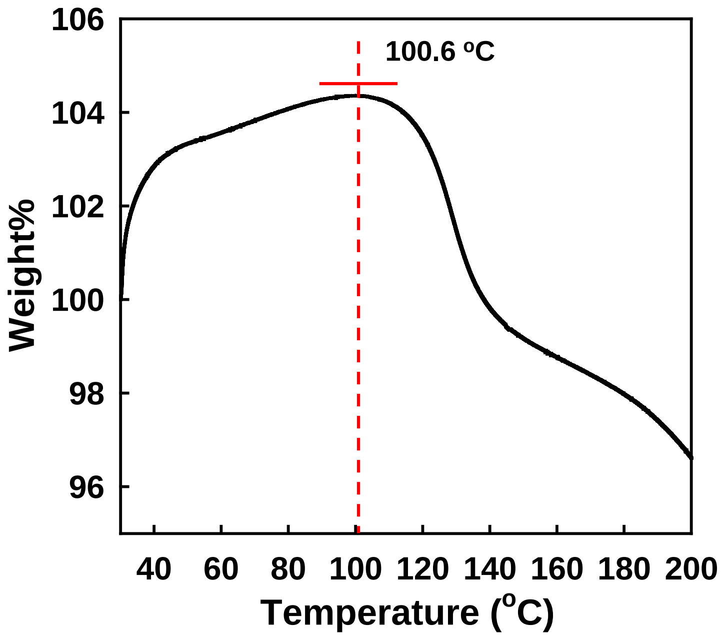

Figure S7. Trace of TGA with flowing 100% CO_2 gas (1 ^{o}C min^{-1}) onto $\mathbf{MOF_{NH2}}$. Equilibrium of CO_2 adsorption and desorption occurs at 100.6 ^{o}C.

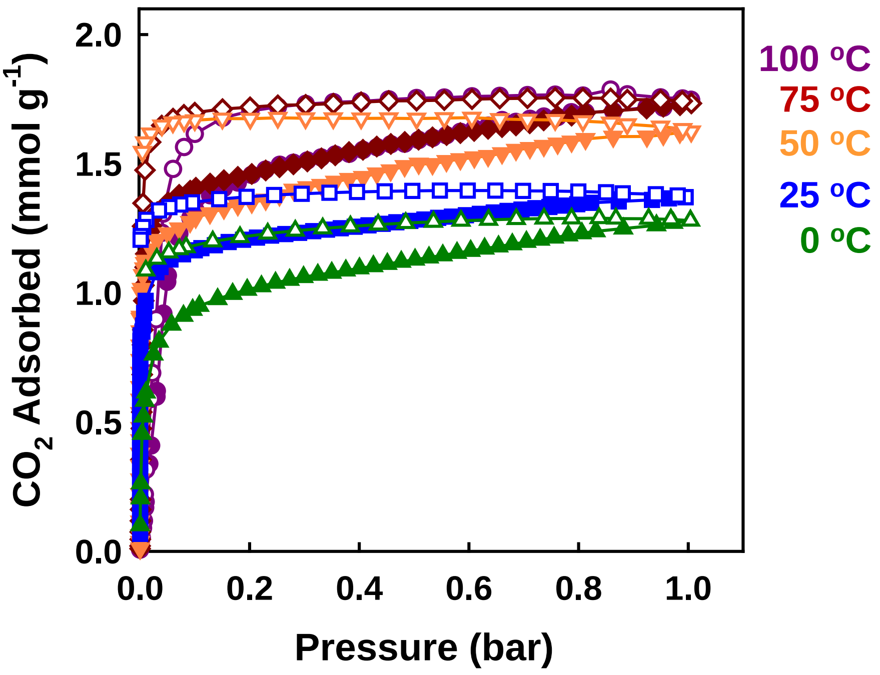

Figure S8. CO_2 gas sorption isotherms of **$MOF_{NH2:powder}$** at 0-100 ^{o}C.

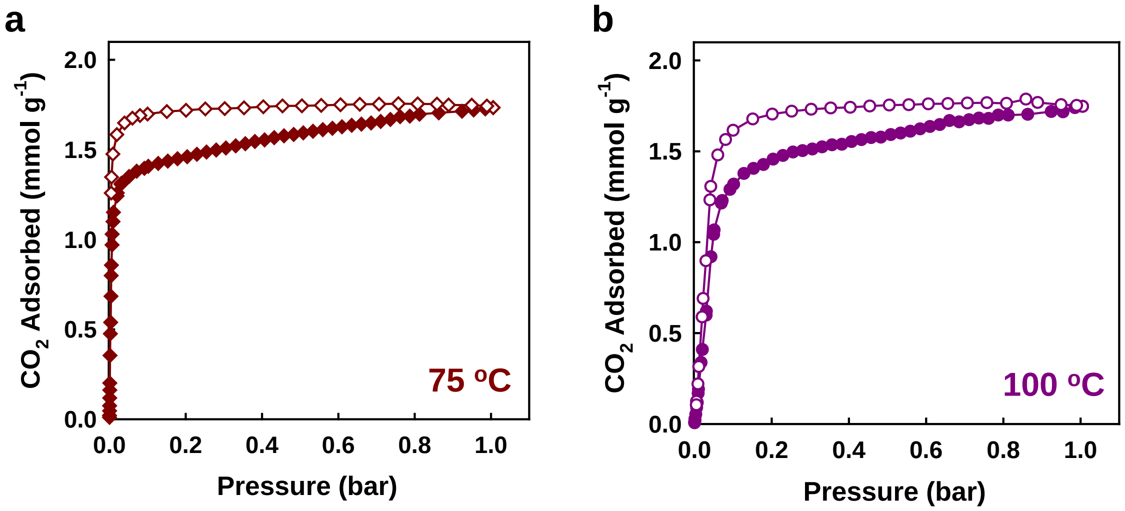

Figure S9. CO_2 gas sorption isotherms of **$MOF_{NH2:powder}$** (a) 75 ^{o}C (b) at 100 ^{o}C

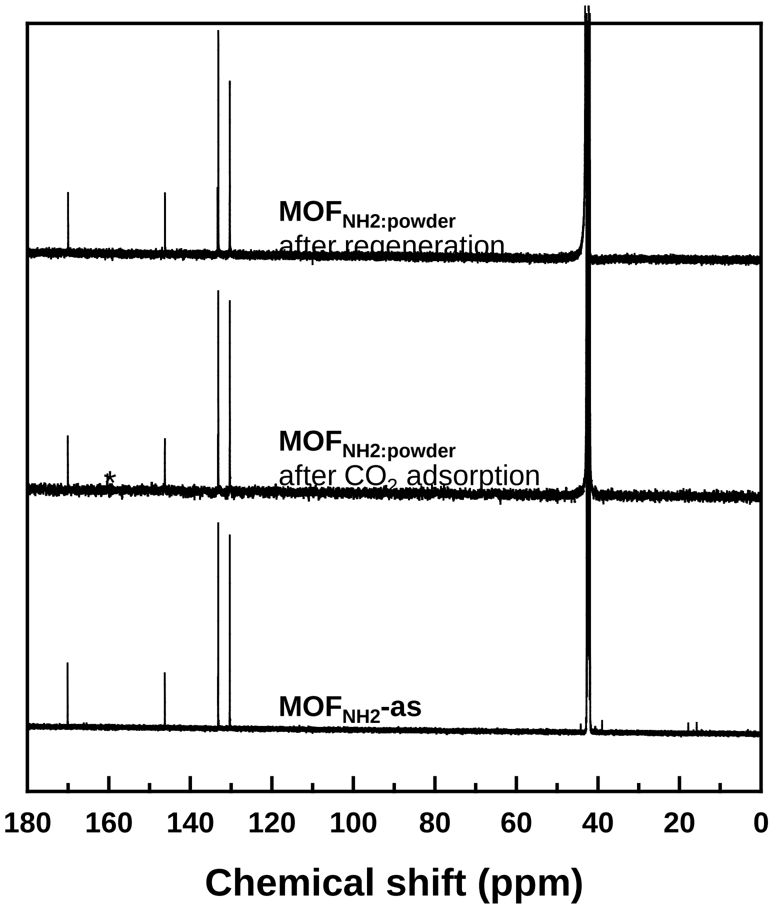

Figure S10. 600 MHz ^{13}C NMR spectra of (a) **MOF_{NH2}-as,** and (b) **$MOF_{NH2:powder}$** after CO_2 adsorption at 75 ^{o}C and (c) the MOF after regeneration at 120 ^{o}C under vacuum for 1 h. The peak at 160.5 ppm (marked with the asterisk) was identified as being due to the formation of carbamate by the chemical interaction between the amine groups and CO_2 molecules. The peaks at 130, 133, 146, and 170 ppm represent the BPDC ligand.

Calculation of isoseric heat of adsorption (Q_{st})

A single-site Langmuir-Freundlich equation (eq 1) was employed to fit the experimental data for CO_2 adsorption in **MOF$_{\textbf{NH2:powder}}$**.

$$q = \frac{q_{sat} \cdot b \cdot p^{\alpha}}{1 + b \cdot p^{\alpha}} \qquad (1)$$

q is the CO_2 uptake (mmol g^{-1}), p is the pressure (bar), q_{sat} is the saturated capacity (mmol g^{-1}), b is the Langmuir-Freundlich constant and α is the Langmuir-Freundlich exponent.

Utilizing single-site Langmuir adsorption model, the exact pressures was obtained with the CO_2 adsorption data at 50 and 75 ^{o}C. The Clausius-Clapeyron formula (eq 2) was used to calculate the isosteric heats of adsorption (Q_{st}).

$$(\ln p)_q = \left(\frac{Q_{st}}{R}\right)\left(\frac{1}{T}\right) + C \qquad (2)$$

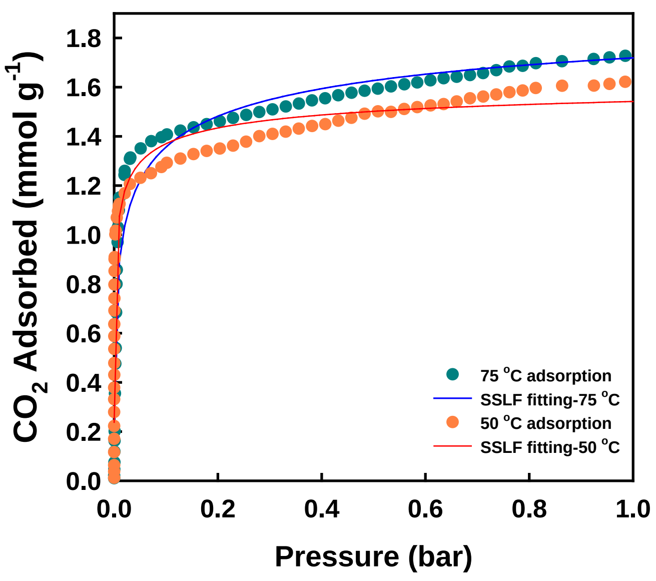

	50 ^{o}C	75 ^{o}C
q_{sat}	1.68	2.09
b	10.88	4.57
α	0.40	0.40

Figure S11. Experimental CO_2 adsorption data in **MOF$_{\textbf{NH2:powder}}$** at 50 and 75 ^{o}C., and corresponding single-site Langmuir-Freundlich (SSLF) isothem fit.

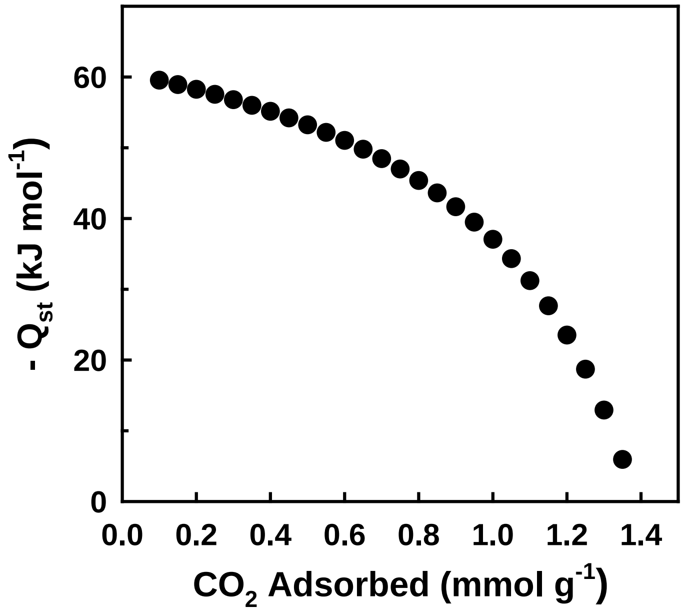

Figure S12. Isosteric heat of adsorption for CO_2 for **MOF$_{NH2:powder}$**, as calculated from fits to the gas adsorption data collected at 50 and 75 ^{o}C.
